# Novel Role of Lck in Leptin-Induced Inflammation and Implications for Renal Aging

**DOI:** 10.14336/AD.2019.0218

**Published:** 2019-12-01

**Authors:** Dae Hyun Kim, June Whoun Park, Hyoung Oh Jeong, Bonggi Lee, Ki Wung Chung, Yujeong Lee, Hee Jin Jung, Min Kyung Hyun, A Kyoung Lee, Byeong Moo Kim, Byung Pal Yu, Hae Young Chung

**Affiliations:** ^1^Department of Pharmacy, College of Pharmacy, Pusan National University, Gumjung-gu, Busan 46241, Korea.; ^2^Korean Medicine (KM)-Application Center, Korea Institute of Oriental Medicine (KIOM), Daegu 41062, Korea.; ^3^Department of Pharmacy, College of Pharmacy, Kyungsung University, Nam-gu, Busan 48434, Korea.; ^4^Department of Physiology, The University of Texas Health Science Center, San Antonio, TX 78229, USA

**Keywords:** Aging, Leptin, Lck, STAT3, NF-κB

## Abstract

Aging is associated with increased fat mass and elevated serum leptin levels (hyperleptinemia), causing proinflammation in the kidneys where it plays a primary role in the removal of endogenous leptin from the circulation. Lymphocyte-specific kinase (Lck) is a positive regulator of inflammatory signaling and a potential treatment target for age-related diseases, but its role in leptin signaling is unknown. Here, we investigated how Lck influences hyperleptinemia-induced inflammation in kidney tissues from 6- and 21-month-old rats. Results indicate that Lck expression and activation increased significantly in aged rat kidneys, especially at renal tubules. Furthermore, we identified interactions between Lck and short leptin-receptor isoforms, suggesting that Lck is a protein tyrosine kinase regulating leptin signaling. We further investigated whether increased Lck expression in renal tubular epithelial cells and macrophage infiltration are associated with leptin-induced inflammation. We then demonstrated that leptin activates Lck and proinflammatory transcription factors (STAT3 and NF-κB), while Lck knockdown modulates the expression of both transcription factors. Collectively, these data implicate that Lck leads to development of leptin-induced renal inflammation during aging. Inhibition of this protein tyrosine kinase may therefore be an appropriate therapeutic option for protection against age-related hyperleptinemia.

Chronic low-grade systemic inflammation is a symptom of both aging and obesity [1]. Indeed, rat experiments demonstrated that an ad libitum diet and restricted movement as a reflective of modern human lifestyle result in a metabolic profile that is similar to that of aging humans [2], with elevated fat mass, inflammatory parameters, and leptin levels [3, 4]. In particular, excess leptin (hyperleptinemia) plays a role in promoting inflammation. Thus, the protein is considered a primary molecular target for research linking chronic inflammation and age-related obesity.

Leptin is found throughout the body and is an adipocyte-secreted pleiotropic hormone of 16 kDa hormone with numerous important roles, including regulation of inflammatory immune responses [5]. Its molecular structure is similar to that of the proinflammatory interleukin-6 cytokine family, including interleukin-6, interleukin-11, interleukin-12 (IL-6, 11, 12), leukemia inhibitory factor, and oncostatin-M [6]. Chronic leptin elevation induces inflammation in the kidneys, leading to pathologies and potentially accelerating aging [7]. A possible mechanism for leptin action and influence in renal disease progression is by regulating macrophage infiltration and proinflammatory action [8].

Several proteins are likely key for proper leptin function. Leptin receptor (LepR) is a class I cytokine with six spliced isoforms (LepRa-f). Of these, only LepRb is a long isoform with a 301-amino-acid intracellular domain, whereas LepRa, LepRc, and LepRd have 34-, 32-, and 40-amino acid intracellular domains, respectively [9]. Although short isoforms contain only the box1 motif, they are still able to interact with Janus kinases (JAK) and activate certain signaling pathways [10-12]. Kidneys express significantly more short isoforms [13], and although their precise biological functions are unclear, some data suggest an involvement of leptin’s inflammatory effects [14].

Another important protein is lymphocyte-specific kinase (Lck), a Src family tyrosine kinase broadly expressed in multiple tissues and cells. Several members of this family, such as c-Src and Fyn, are already known to be involved in JAK2-independent leptin signaling [15]. Previously, we determined that Lck is a key mediator in the aging process, but only when inflammation-related pathways are upregulated [16]. Lck is involved in the release of proinflammatory cytokines and chemokines through activating two inflammatory transcription factors: signal transducer and activator of transcription 3 (STAT3) and nuclear factor-κB (NF-κB) [17-19]. These two transcription factors have a synergistic effect that can lead to chronic inflammation and cancer [20, 21]. However, the exact mechanism of Lck action is unknown, but may be related to leptin, which exerts proinflammatory effects via STAT3 and NF-κB pathways [22].

This study aimed to investigate that Lck is a key mediator of hyperleptinemia-induced inflammatory response in aged rat kidney. We focused on a novel mechanism of Lck-STAT3/NF-κB pathways implicated in leptin-induced renal inflammation. We evaluated interactions between Lck and leptin receptors, as well as STAT3 and NF-κB activation in young and old male rats. We then analyzed rat kidney epithelial cells and mouse macrophages to understand how Lck interacts with the STAT3 and NF-κB pathways and modulate leptin-induced inflammation.

## MATERIALS AND METHODS

### Materials

Antibodies against Lck, p-Lck (Tyr394), STAT3, p65, p-p65 (Ser536), LepR, GAPDH, and TFIIB were obtained from Santa Cruz Biotechnology (Dallas, TX, USA). The antibody against p-STAT3 (Tyr705) was obtained from Cell Signaling Technology (Danvers, MA, USA). Anti-rabbit IgG-horseradish peroxidase-conjugated antibody and anti-mouse IgG-horseradish peroxidase-conjugated antibody were obtained from Santa Cruz Biotechnology. Polyvinylidene difluoride (PVDF) membranes were obtained from Millipore Corporation (Bedford, MA, USA). Dokdo-MARK^TM^ protein size marker was obtained from ElpisBiotech (Daejeon, Korea). Rat and murine recombinant leptins were purchased from PeproTech (Rocky Hill, NJ, USA). Lck inhibitor (7-cyclopentyl-5-(4-phenoxyphenyl)-7H-pyrrolo [2,3-d]pyrimidin-4-ylamine) was obtained from Sigma-Aldrich (St. Louis, MO, USA). The highest available grade was selected for all materials.

### Animal experimental procedures

Young (6 months) and old (21 months) male Sprague Dawley (SD) rats were purchased from the Samtako (Osan, Kyoungki-do, Korea). Their diet composition was as follows: 14 g casein, 0.18 g L-cysteine, 46.57 g corn starch, 15.5 g dextrinized corn starch, 10 g sucrose, 5 g fiber, 4 g soybean oil, 3.5 g AIN-93 mineral mix, 1 g AIN-93 vitamin mix, 0.25 g choline bitartrate, and 0.8 mg TBHQ. Each group contained six rats, all housed under pathogen-free conditions, with a 12 h light/dark cycle at 23 ± 1°C and 50 ± 5% relative humidity. Young and old rats were allowed to eat chow *ad libitum* for 2 weeks before euthanasia. Kidneys were collected for biochemical analysis and western blotting. All protocols were reviewed and approved beforehand by the Pusan National University-Institutional Animal Care and Use Committee (PNU-IACUC; Approval Number 2013-0409).

For leptin (450-31, PeproTech) administration, individually caged 7-week-old male mice (n = 5 per group) on chow diet were first acclimated for 1 d after i.p. injections of Lck inhibitor (Sigma-Aldrich). Over the next 3 d, mice were i.p. injected daily (8:00 AM) with phosphate-buffered saline (PBS) or 1 mg/kg of recombinant mouse leptin. Mice were sacrificed at 1:00 PM, 5 h after the eighth injection on day 4. Tissues of interest were snap-frozen in liquid nitrogen immediately after resection and stored at -80°C.

### Cell culture

A normal rat-kidney tubular epithelial cell line (NRK52E) and a mouse macrophage cell line (J774a.1) (American Type Culture Collection, Manassas, VA, USA) were grown in Dulbecco’s modified eagle medium (DMEM, HyClone Laboratories, Logan, UT, USA) and Roswell Park Memorial Institute 1640 (RPMI 1640, Welgene, Gyeongsan-si, Gyungsangbuk-do, South Korea), respectively. The latter contained 2 mM L-glutamine, 100 units/mL penicillin, 100 μg/mL streptomycin, and 10% heat-inactivated fetal bovine serum (FBS, HyClone). Cells were cultured for 1 d at 37°C in a humidified atmosphere containing 5% CO_2_/95% air. Subsequently, media were replaced to remove non-adherent cells or cell debris. Cells were then washed with PBS before adding 1 mL of ice-cold PBS. Pellets were centrifuged at 900 ×*g* at 4°C for 5 min.

### Protein isolation from tissues and cells

All solutions and samples were maintained at 4°C. Approximately 100 mg of frozen kidney tissue was mixed for 30 s in a tissue homogenizer with 1 mL of homogenate buffer, containing 100 mM Tris, 20 mM β-glycerophosphate, 20 mM NaF, 2 mM sodium orthovanadate, 1 mM EDTA, 0.01 mM dithiothreitol (DTT), 0.5 mM phenylmethylsulfonyl fluoride (PMSF), 1 μM pepstatin, and 80 mg/L trypsin inhibitor (pH 7.4). After being kept on ice for 20 min, 125 μL of 10% Nonidet P-40 (NP-40) solution was added to the tissue samples, mixed for 15 s, and centrifuged at 14,000 ×*g* at 4°C for 5 min. The supernatant was used as the cytosol fraction. Pellets were washed once with 400 μL homogenate buffer containing 50 μL of 10% NP-40 and centrifuged a second time. Pellets were then suspended in 100 μL of buffer containing 50 mM KCl, 300 mM NaCl, 0.1 mM EDTA, 10% (v/v) glycerol, 0.01 mM DTT, 20 mM β-glycerophosphate, 20 mM NaF, 2 mM sodium orthovanadate, 1 mM EDTA, 0.5 mM PMSF, 1 μM pepstatin, and 80 mg/L trypsin inhibitor. After incubating on ice for 30 min, samples were centrifuged a at 14,000 ×*g* at 4°C for 10 min. Nuclear proteins were collected from the supernatant and stored at -80°C. Protein concentration was measured with a bicinchoninic acid (BCA) assay using bovine serum albumin (BSA) as the standard.

Cells were washed with 1X PBS before the addition of more ice-cold PBS (1 mL), followed by centrifugation at 1,000 ×*g* at 4°C for 5 min. Pellets were suspended in buffer (10 mM Tris at pH 8.0, 1.5 mM MgCl_2_, 1 mM DTT, 0.1% Nonidet-40, and protease inhibitors), incubated on ice for 15 min, and then centrifuged again at 14,000 ×*g* at 4°C for 5 min. The supernatant was used as the cytosolic fraction. Pellets were suspended in 10 mM Tris (pH 8.0) containing 50 mM KCl, 100 mM NaCl, and protease inhibitors, before a 30 min incubation on ice, followed by centrifugation at 14,000 ×*g* at 4°C for 30 min. The resultant supernatant was used as the nuclear fraction.

### Gene expression analysis with real-time PCR

Kidney tissue samples and cells were separately treated with a bead homogenizer (TissueLyser II, Qiagen, Hilden, Germany) in the presence of RiboEx (GeneAll, Seoul, Korea). Chloroform (1/10 volume) was added before samples were shaken vigorously, incubated on ice for 5 min, and then centrifuged at 16,810 ×*g* at 4°C for 15 min. After discarding the supernatant, the RNA pellet was precipitated with isopropanol, washed with 75% ethanol, dried, and dissolved in diethyl pyrocarbonate-treated water. Complementary DNA was synthesized using amfiRivert platinum cDNA synthesis master mix (GenDEPOT, Barker, TX, USA) for use as a template in a real-time PCR, performed with SYBR Green real-time master mix (Geneall, Seoul, Korea). Real-time PCR and data analyses were performed with the CFX Connect System (Bio-Rad Laboratories Inc., Hercules, CA, USA). Primer sequences used to detect mRNA expression are provided in Table 1.

**Table 1:** Primer sequences for qPCR

Gene	Forward (5′-3′)	Reverse (3′-5′)
Rat		
**Lck**	CGT GTG TGA AAA CTG CCA CT	ACC CTT TTC AAA GCC CAA GT
**IL-6**	GAA GTA GGG AAG GCA GTG GC	TCA TTC TGT CTC GAG CCC AC
**MCP-1**	AGC ATC CAC GTG CTG TCT C	GAT CAT CTT GCC AGT GAA TGA G
**ACTA2**	TTG TCC ACC GCA AAT GCT TC	AAG GCG CTG ATC CAC AAA AC
**Col1α**	CGT CGT GCC TAG CAA CAT GC	AGT TCC CAG TAA GAC CAG GG
**Fn**	ATT CCA ATG GTG CCT TGT GC	TGC CGC ACC ATT TCA TGT TG
**Col3α1**	ATC AAA CAC GCA AGG CCA TG	AAG CAA ACA GGG CCA ATG TC
**Col4α**	GGT GAT TGT GGT GGC TCT GG	CGT GTC CCT TTC GTT CCA GG
**Col11α**	CCA TCT CAA CCC TCG TCT TGA CT	GGG CAC TGA TCT GGG CTT CCT
Mouse		
**Lck**	CAC GGA TGA CAG CTC TGA AA	ATG GAG AAC GGG AGC CTA GT
**IL-6**	CCA CGA TTT CCC AGA GAA CA	TTG CCT TCT TGG GAC TGA TG
**MCP-1**	CTT CTT GGG GTC AGC ACA GA	CCA GCA AGA TGA TCC CAA TG

### Western blotting

Homogenized kidney samples (10 μg of protein from each nuclear fraction and 20 μg of protein from each cytosolic fraction) were boiled for 5 min in gel-loading buffer (0.125 M Tris-HCl, pH 6.8, 4% SDS, 10% 2-mercaptoethanol, and 0.2% bromophenol blue) at a 1:1 volume ratio. Equivalent total protein samples were separated through 10% SDS-polyacrylamide gel electrophoresis (PAGE) and then transferred to PVDF membranes at 90 V for 1 h using a wet transfer system. Membranes were immediately placed in a blocking buffer (10 mM Tris [pH 7.5], 100 mM NaCl, 0.1% Tween 20, and 5% non-fat milk) at room temperature for 30 min and then incubated with specific primary antibodies (dilution: 1:1000) at 4°C overnight. After a 30 min wash in TBS-T buffer, membranes were incubated with either anti-rabbit or anti-mouse IgG-horseradish peroxidase-conjugated secondary antibody (Santa Cruz Biotechnology, dilution: 1:10000) at 25°C for 1 h, and then washed again in TBS-T buffer for 40 min. Each antigen-antibody complex was visualized using ECL Western Blotting Detection Reagents and detected through chemiluminescence with Sensi-Q 2000 (Lugen Sci., Gyeonggido, Korea).

### Immunoprecipitation

Kidney homogenates were immunoprecipitated in a buffer containing 40 mM Tris-HCl (pH 7.6), 120 mM NaCl, 20 mM glycerophosphate, 20 mM NaF, 2 mM sodium orthovanadate, 5 mM EDTA, 1 mM PMSF, 0.1% NP-40, leupeptin (2 µg/mL), aprotinin (1 µg/mL), and pepstatin A (1 µg/mL). Kidney-homogenate aliquots were centrifuged at 12,000 ×*g* at 4°C for 15 min, incubated overnight at 4°C with corresponding antibodies, and then incubated for another night at 4°C with 50% protein A-agarose slurry. After washing with immunoprecipitation buffer three times, immunoprecipitated proteins were analyzed with SDS-PAGE and western blotting (see “Western blotting”).

### Small interference RNA transfection

To silence Lck, small interference RNAs (siRNA) were synthesized (IDT, Coralville, IA, USA) and transfected into NRK52E cells. The following Lck (NM_001100709) siRNA sequences were used: forward, 5′-AGU CUG AAG CAA GGG AGU AUG UCTC-3′; reverse, 3′-UUU CAG ACU UCG UUC CCU CAU ACA GAG-5′. Non-targeting scrambled RNA (IDT) was used as a negative control. One day before transfection, cells were seeded in 60 mm plates and incubated for 24 h with growth medium. Cells were 50-60% confluent at transfection and treated with 40 nM scrambled or Lck siRNA. Transfection was performed using Lipofectamine 3000 complexes in Opti-MEM (Invitrogen, Carlsbad, CA, USA) without serum, following the manufacturer’s protocol. After transfection, cells were incubated for 48 h in growth medium without antibiotics.

### Immunohistochemistry analysis

Kidney tissues were fixed in 10% formalin at room temperature. Paraffin-embedded specimens were sectioned (5 μm), deparaffinized, and stained with 3,3′-diaminobenzidine (DAB) for visualizing Lck (Santa Cruz Biotechnology) or CD68 antibodies (Santa Cruz Biotechnology). Sections were also processed with hematoxylin and eosin staining for light microscopic evaluation. Stained tissue sections were examined under a Motic® AE30/31 inverted microscope (Motic Incorporation, Xiamen, China), and five to six images (200×) were taken per section. Sections were visualized with periodic acid Schiff (PAS) staining to determine renal glomerular and tubular damage, with observer’s blind to experimental conditions scoring the images.

### Sample preparation for Illumina HiSeq 2000 sequencing

After euthanasia through CO_2_ inhalation, kidney cortices from six SD rats were quickly removed, immediately frozen in liquid nitrogen, and stored at -80°C. Total RNA was then extracted from each sample using the miRNeasy Mini Kit (Qiagen). An RNA sequencing library was generated using a TruSeq RNA sample preparation kit, separating mRNA from total RNA using Oligo(dT) beads and chemical fragmentation following the manufacturer’s protocol (Illumina, San Diego, CA, USA). End repair, 3′-end adenylation, and sequencing adapter ligation were performed on cDNA synthesized from fragmented mRNA, followed by purification with magnetic beads and PCR amplification. The amplified library was purified, quantified, and then used to prepare templates for generating 99 bp paired-end sequencing reads in the Illumina HiSeq 2000 platform.

### Serum analysis

Blood samples were collected from male rats (n = 6) and mice (n = 5) after euthanasia. The Mouse/Rat Leptin Quantikine ELISA kit (R&D Systems, Oxford, UK) was used to determine serum leptin concentrations.

Serum urea and creatinine levels were quantified using a commercially available kit from Biosystems (Barcelona, Spain).

### Immunofluorescence analysis

Rats were euthanized after a 16 h fast. Kidneys were retrieved and fixed in 4% paraformaldehyde for 16 h at 4°C, followed by incubation in 30% sucrose overnight at 4°C. Cyrosections (5 μm) were co-immunostained using a mixture of rabbit Lck (sc-13, Santa Cruz Biotechnology) and mouse anti-CD68 (sc-20060, Santa Cruz Biotechnology). Additional co-immunostaining experiments were performed using a mixture of rabbit Lck (sc-13, Santa Cruz Biotechnology) and mouse anti-LepR (sc-8391, Santa Cruz Biotechnology). After washing with PBS buffer containing 0.5% Tween 20, sections were incubated for 2 h in the dark at room temperature, with a mixture of two secondary antibodies [Alexa Fluor-488 goat anti-rabbit IgG (H+L) antibody (Invitrogen) and Rhodamine Red^TM^-X goat anti-mouse IgG (H+L) antibody (Jackson ImmunoResearch, West Grove, PA, USA)] in 1% BSA. Sections were then counterstained for 5 min with 1 μg/mL DAPI, mounted, and stored in the dark at 4°C. Confocal images were obtained using an FV10i FLUOVIEW Confocal Microscope (Olympus, Tokyo, Japan).

**Figure 1.**
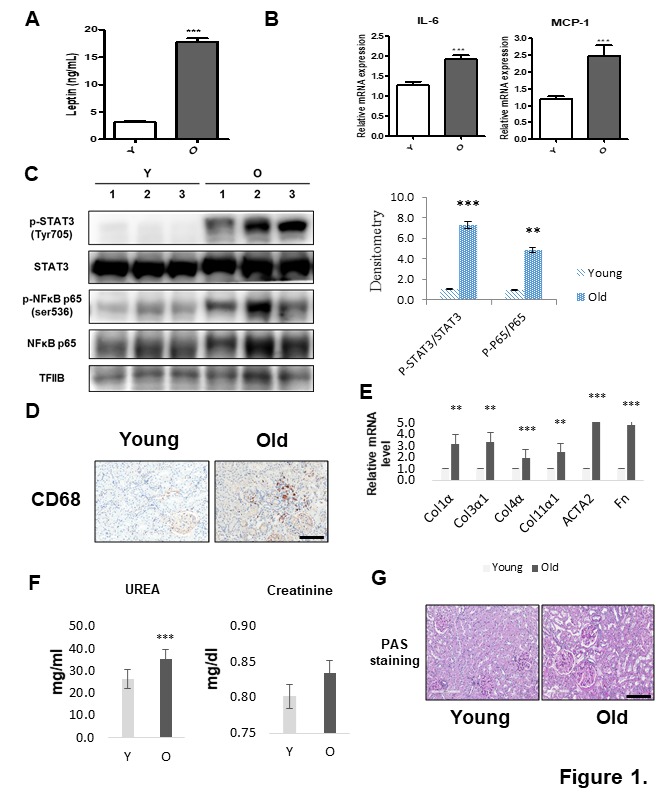
Hyperleptinemia and renal inflammation in aged kidney. (A) Serum leptin was determined by ELISA. Data were expressed as mean ± SEM. Statistical results of one-factor ANOVA and Bonferroni tests: ^***^p < 0.001 vs. young rats. (B) Real-time PCR of IL-6 and MCP-1 mRNA levels, normalized to GAPDH. (C) Western blots detected STAT3 and NF-κB in nucleus fraction of young and old kidney issues (from 6-month-old and 21-month-old rats, respectively), based on phosphorylation at Tyr705 and Ser536, respectively. TFIIB blots verified the amount of protein loaded. Bars in densitometric data represent means ± SE (n = 6), and significance was determined using an unpaired *t* test: **p < 0.01; ***p < 0.001 vs. young. (D) Immunohistochemistry of representative macrophage recruitment. Kidney sections were stained with a specific antibody (CD68) against macrophages. (E) Results of qPCR revealing extracellular matrix gene expression. *p < 0.05 vs. young. (F) Serum urea and creatinine levels were measured to evaluate kidney function. *p < 0.05 vs. young. (G) Aging-related renal damage was visualized with PAS staining. Scale bar = 200 μm.

**Figure 2.**
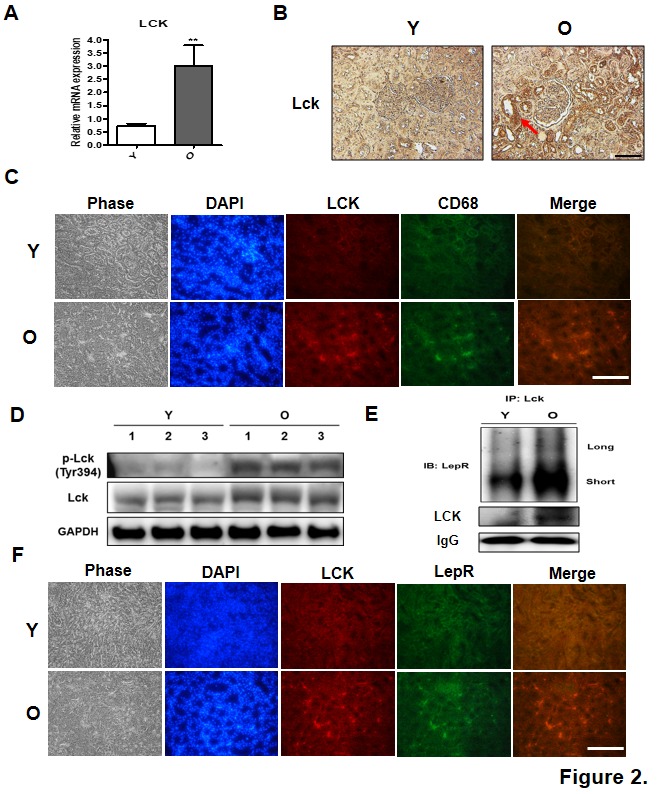
Changes in Lck expression and activation in renal aging. (A) Real-time PCR determined Lck expression in young and old rat kidney tissues. Data were expressed as mean ± SEM. Statistical results of one-factor ANOVA and Bonferroni tests: ^**^p < 0.01 vs. young rats. (B) Distribution of Lck in young and old rat kidneys was evaluated by DAB immunostaining. (C) Lck and macrophage marker CD-68 were detected using two primary antibodies. Lck antibody and CD-68 antibody were simultaneously administered to young and old kidney paraffin sections for double immunofluorescence staining. Tissue sections were counterstained with DAPI. After staining, fluorescence was observed using confocal microscopy. Scale bar = 100 μm. (D) Western blots measured total Lck protein level and phosphorylation at Tyr394. GAPDH blots clarified the amount of protein loaded. (E) Immunoprecipitation and western blots determined Lck interactions with LepR. Lck binding to LepR short isoforms in rat kidney increases with age. (F) Lck and LepR colocalize in the kidney. Frozen sections of aged kidney tissues were coimmunostained with anti-Lck and anti-LepR antibodies. Cell nuclei were stained with DAPI. Scale bar = 100 μm.

### Statistical analysis

One-way analysis of variance (ANOVA) was used to analyze differences among three or more groups. Differences in the means of individual groups were assessed using Bonferroni’s *post hoc* test. Results were considered statistically significant at p-values < 0.05. Analysis was performed using GraphPad Prism 5 software (La Jolla, CA, USA).

**Figure 3.**
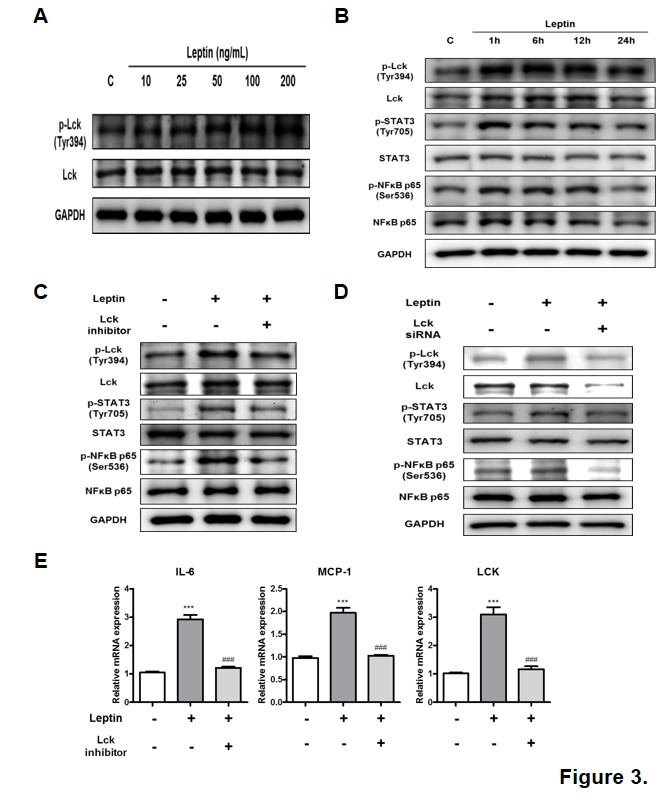
Leptin-induced inflammation via activation of Lck-STAT3/NF-κB signaling pathway in NRK52E. (A) NRK52E cells were treated with leptin at different concentrations from 0 to 200 ng/mL and incubated for 30 min. Control was treated with vehicle only. Western blotting was performed to detect the activation of Lck represented by phosphorylation at Tyr 394 of Lck. (B) NRK52E cells were treated with 200 ng/mL of leptin and incubated for 0, 1, 6, 12, and 24 h. Western blotting was performed to detect activation of Lck, STAT3, and NF-κB signaling pathway. GAPDH blots clarified the amount of protein loaded. One representative blot of each protein is shown from three experiments that yielded similar results. (C) NRK52E cells were pretreated with Lck-specific inhibitor (100 nM) for 30 min and then treated with 200 ng/mL leptin for 60 min. (D) NRK52E cells were treated with Lck siRNA for 48 h and then treated with 200 ng/mL leptin for 60 min. Leptin-induced activation of Lck, STAT3, and NF-κB was downregulated by Lck inhibitor and siRNA. GAPDH blots clarified the amount of protein loaded. One representative blot of each protein is shown from three experiments that yielded similar results. (E) NRK52E cells were pretreated with Lck-specific inhibitor (100 nM) for 30 min and then treated with 200 ng/mL leptin for 16 h. Real-time PCR was performed to detect mRNA levels of IL-6, MCP-1, and Lck. Data were expressed as mean ± SEM. Statistical results of one-factor ANOVA and Bonferroni tests: ^***^p < 0.001 control vs. leptin; ^###^*p <* 0.001 leptin vs. Lck inhibitor.

**Figure 4.**
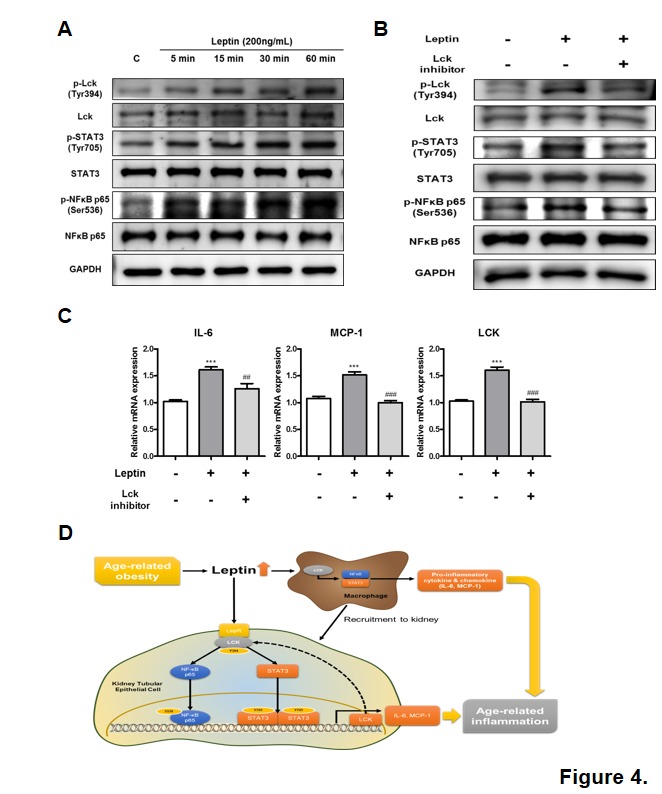
Leptin-induced inflammation via activation of Lck-STAT3/NF-κB signaling pathway in J774a.1. (A) J774a.1 cells were treated with 200 ng/mL of leptin and then incubated for 5, 15, 30, and 60 min. Control was treated with vehicle. Western blotting was performed to detect activation of Lck, STAT3, and NF-κB signaling pathway. GAPDH blots clarified the amount of protein loaded. One representative blot per protein is shown from three experiments that yielded similar results. (B) J774a.1 cells were pretreated with Lck-specific inhibitor for 30 min and then treated with 200 ng/mL leptin for 60 min. Western blotting detected activation of Lck, STAT3, and NF-κB signaling pathway. GAPDH blots clarified the amount of protein loaded. One representative blot per protein is shown from three experiments that yielded similar results. (C) J774a.1 cells were pretreated with Lck-specific inhibitor for 30 min and then treated with 200 ng/mL leptin for 16 h. Real-time PCR was performed to detect the mRNA levels of IL-6, MCP-1, and Lck. Data were expressed as mean ± SEM. Statistical results of one-factor ANOVA and Bonferroni tests: ^***^p < 0.001 control vs. leptin; ^##^*p <* 0.01, ^###^*p <* 0.001 leptin vs. Lck inhibitor. (D) Possible mechanisms of leptin-Lck signaling in renal aging. Aging-induced hyperleptinemia directly induces inflammation in renal tubular epithelial cells by binding LepR short forms to activate Lck-STAT3/NF-κB signaling pathways and increasing expression of proinflammatory genes (IL-6, MCP, and Lck). In addition, Lck may play a role in leptin directly or indirectly triggering macrophage infiltration of aged kidneys, further inducing inflammation.

## RESULTS

### Increased serum leptin and renal inflammation in aging

Serum leptin increased by over five-fold in old rats compared with young rats (Fig. 1A), implying that aging results in leptin resistance leading to hyperleptinemia. Consistent with this outcome, aged kidney tissue also had significantly higher mRNA expression level of IL-6 and the chemokine MCP-1 (Fig. 1B).

We examined STAT3 and NF-κB activation through evaluations of Tyr705 and Ser536 phosphorylation, respectively, as well as their nuclear protein levels. Western blots revealed a slight increase in TFIIB levels among aged kidney tissues. Additionally, Tyr705 and Ser536 proteins increased in nuclear fractions of aged kidney (Fig. 1C). Aging kidney sections were then stained with a CD68 antibody to determine the expression levels of macrophages in kidney inflammation. Immuno-histochemistry demonstrated increased macrophage infiltration in old rats (Fig. 1D).

We also verified age-related renal functional changes and epithelial damage. Extracellular matrix gene (Col1α, Col3α1, Col4α, Col11α1, ACTA2, and Fn) expression levels were significantly increased in aged rat kidney (Fig. 1E). Additionally, serum urea was significantly increased in old rats (Fig. 1F), whereas level of creatinine did not change. Finally, aged rat kidneys exhibited a significant increase in glomerular PAS staining, indicative of heightened matrix accumulation (Fig. 1G).

### Increased Lck expression and interaction with leptin receptor in aged kidney

Sequencing results revealed that Lck mRNA expression in aged kidney was upregulated by over three-fold compared with young kidney (Table 2), a result confirmed using real-time PCR (Fig. 2A). Immunohistochemical staining showed that Lck distribution in renal tubules also increased in old kidney (Fig. 2B). Moreover, immunofluorescence revealed elevated colocalization of CD-68 and Lck in aged rat kidneys (Fig. 2C).

Western blot analyses of kidney cytosol fractions showed higher phosphorylation at Tyr394 (indicating Lck activation) in old rats than young rats (Fig. 2D). Collectively, these results suggest that Lck upregulation in renal tubular epithelial cells (leptin-absorption site) of aged kidneys is linked to age-related hyperleptinemia.

Immunoprecipitation analyses showed that Lck directly interacted with only LepR short forms, a relationship that increased in old rats (Fig. 2E). Finally, immune-fluorescence analysis found that Lck and LepR colocalize in aged rat kidneys (Fig. 2F).

**Table 2:** Lck expression in kidney tissues of young and old rats determined by Illumina HiSeq 2000 sequencing.

Genes	Old_Young	Kidney
Lck	3.387732589	lymphocyte-specific protein tyrosine kinase

### Leptin-induced activation of Lck, STAT3, and NF-κB in kidney tubular epithelial cells

Leptin treatment to NRK52E cells increased Lck phosporylation at Tyr394 (Fig. 3A). Additionally, western blotting showed an increase in Lck phosphorylation at Tyr394 shortly after 1 h of leptin treatment that was maintained until 12 h, before gradually decreasing by 24 h (Fig. 3B). Leptin treatment also increased total Lck protein levels between 6 to 12 h. Consistent with Lck activation, leptin treatment upregulated phosphorylation of STAT3 at Tyr705 and NF-κB at Ser536 between 1 to 12 h (Fig. 3B). Taken together, these results demonstrate that age-related hyperleptinemia activated Lck-STAT3/NF-κB signaling pathways, increasing Lck expression in rat kidney tubule epithelial cells.

### Inhibition or knockdown of Lck in kidney tubular epithelial cells downregulates leptin-induced inflammation

When NRK53E cells were treated with Lck inhibitor, we observed a downregulation of leptin-mediated increases in Lck phosphorylation at Tyr394 (Fig. 3C). Phosphorylation of STAT3 at Tyr705 and NF-κB at Ser536 was also downregulated. To confirm results from Lck inhibitor experiments, we used siRNA knockdown of Lck and demonstrated an abrogation of leptin-induced STAT3 and NF-κB phosphorylation at Tyr705 and NF-κB, respectively (Fig. 3D). Taken together, these results indicate that increased expression and activation of Lck correlate with activation of STAT3 and NF-κB in response to leptin.

Leptin treatment of NRK52E cells increased IL-6 and MCP-1 mRNA levels by two- to three-fold (Fig. 3E). Moreover, pretreatment with Lck inhibitor significantly downregulated IL-6, MCP-1, and Lck mRNA under leptin stimulation (Fig. 3E). These results indicate that increased expression and activation of Lck in renal tubular epithelial cells, driven by hyperleptinemia in aging, induced renal inflammation via activation of STAT3 and NF-κB.

### Attenuation of leptin-induced inflammation by modulation of Lck in macrophages

Increased infiltration of macrophages in the kidney is one of the well-known features of aging. To investigate the role of Lck in leptin signaling in macrophages, the activation of Lck by leptin treatment was analyzed by western blotting. J774a.1 macrophages were treated with 200 ng/mL of leptin for 5, 15, 30, and 60 min. As compared with vehicle-treated control, leptin-treated macrophages showed increased phosphorylation of Lck at Tyr394, implying that Lck is activated by leptin (Fig. 4A). Phosphorylation of STAT3 at Tyr705 and NF-κB at Ser536 was also increased by leptin treatment with the maximal effect at 60 min (Fig. 4A).

To determine whether Lck activates STAT3 and NF-κB in response to leptin, western blot analysis was performed to examine phosphorylation of STAT3 and NF-κB in J774a.1 cells. J774a.1 cells were pretreated with 100 nM of Lck inhibitor for 30 min and then treated with 200 ng/mL of leptin for 1 h. Consistent with the result from renal tubular epithelial cells, western blotting showed that phosphorylation of Lck at Tyr394, which was increased by leptin treatment, was diminished by treatment with Lck inhibitor (Fig. 4B). Inhibition of phosphorylation of Lck at Tyr394 resulted in down-regulation of phosphorylation of STAT3 at Tyr705 and NF-κB at Ser536 under the stimulation of leptin (Fig. 4B). Collectively, these *in vitro* findings confirmed that modulation of Lck can regulate the leptin-induced activation of STAT3 and NF-κB signaling pathways.

The target gene expression of IL-6, MCP-1, and Lck was further investigated to verify the attenuation of leptin-induced inflammation in macrophages by modulation of Lck. J774a.1 cells were pretreated with vehicle or 100 nM of Lck inhibitor for 30 min and incubated with 200 ng/mL of leptin for 16 h. Real-time PCR was performed to measure the mRNA levels of IL-6 and MCP-1, which were significantly downregulated by Lck inhibitor (Fig. 4C). In addition, the increased mRNA level of Lck by leptin treatment was also diminished by Lck inhibitor (Fig. 4C). Therefore, these data suggest that regulation of Lck in macrophages attenuated inflammation by reducing production of pro-inflammatory cytokine IL-6 and chemokine MCP-1 as well as inhibiting Lck expression.

**Figure 5.**
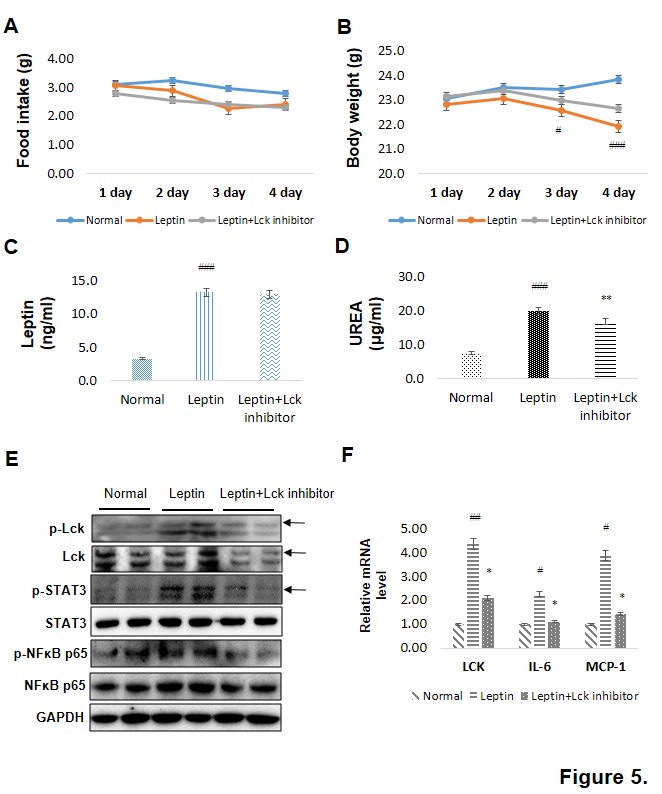
Lck inhibitor suppresses inflammation in kidney tissue. C57BL/6 male mice at 7 weeks of age were pre-injected with Lck-specific inhibitor (10 mg/kg) for 1 day and then treated with PBS vs. leptin (1 mg/kg) via intraperitoneal (i.p.) injection twice a day for 3 days, followed by sacrifice 5 h after the last injection (n? = ?5 per group). (A) Food intake and (B) body weight were measured daily before the dark cycle, and arrows indicate the time points of leptin injections. Data were expressed as mean ± SEM. Statistical results of one-factor ANOVA followed by the Bonferroni test: ^#^*p<*0.05, ^###^*p <* 0.001 vs. normal mice. (C) Serum leptin was measured with ELISA. Data were expressed as mean ± SEM. Results of one-factor ANOVA and Bonferroni tests: ^##^*p <* 0.01 vs. normal mice. (D) Serum urea and creatinine levels were measured to determine kidney function. ^###^*p* < 0.001 vs. normal mice; **p < 0.01 vs. leptin-injected mice. (E) Western blotting was performed to examine the levels of phosphorylation of Lck, STAT3, and NF-κB in the kidney of mice treated with leptin and Lck inhibitor. GAPDH blots clarified the amount of protein loaded. (F) Real-time PCR results of Lck, IL-6, and MCP-1 mRNA levels, normalized with GAPDH. ^#^*p* < 0.05, ^##^*p* < 0.01 vs. normal mice; *p < 0.05 vs. leptin-injected mice.

### Downregulation of leptin-induced inflammation by Lck inhibition in mice

Leptin administration considerably reduced food intake and body weight in mice (Fig. 5A, B). Serum leptin increased in leptin-administered mice by over four-fold compared with normal mice, whereas Lck inhibitor groups exhibited no change (Fig. 5C). We next showed a significant decrease in serum urea level when leptin-injected groups were treated with Lck inhibitor (Fig. 5D). However, creatinine levels did not change (data not shown). At 5 h after the final leptin injection, we detected marked phosphorylation of Lck, STAT3, and NF-κB in the kidney. This activation was suppressed with Lck inhibitor (Fig. 5E). Consistent with these findings, mRNA expression of IL-6 and MCP-1 in kidney decreased significantly by Lck inhibitor treatments (Fig. 5F).

## DISCUSSION

Aging is accompanied by elevated visceral adiposity, serum leptin, and insulin resistance [23], as well as decreased sensitivity to leptin. These changes are closely related to the fact that aging increases the risk of almost all chronic diseases [24]. Existing data suggest that hyperleptinemia plays a major role in the metabolic syndromes and chronic inflammation which are typical physiological changes in aging. Thus, leptin is an interesting molecular target for anti-aging therapies.

This study provides strong evidence for Lck’s role in aging as a pivotal mediator linking energy metabolism and immune response [16]. Increased Lck expression along with upregulation of inflammation pathways, has been shown in kidney, skeletal muscle, heart, and white adipose tissue during aging. Here, we confirmed that Lck is upregulated in aged rat kidney, specifically in tubular epithelial cells (Fig. 2).

Leptin is filtered by the glomerulus and reabsorbed by renal tubular epithelial cells in cortex, where it is retained and metabolized [25, 26]. Quantitative analysis of kidney leptin uptake revealed leptin accumulation in rat renal cortex [27]. Such accumulation suggests that hyperleptinemia in age-related obesity may significantly increase the burden on renal epithelial cells through elevated leptin-induced inflammation. Although our results suggest that this inflammation may be related to leptin binding with LepR short isoform, previous studies show that megalin (gp330/LRP2) is the molecule that many participate in inflammatory response by mediating leptin transport or reabsorption in renal tubules [28]. Nevertheless, we identified interactions between Lck and LepR short isoform in aged kidney in rats (Fig. 2E), suggesting a connection between Lck and heightened inflammation in renal aging. We also observed that Lck modulates leptin-induced activation of STAT3 and NF-κB, thus leading to upregulation IL-6 and MCP-1 production in kidney cells (Fig. 3E). Corroborating our *in vivo* results, leptin-induced increases in Lck expression may amplify inflammatory pathways during renal aging. Taken together, these data imply that leptin induces renal inflammation through upregulation of Lck.

Aged kidneys typically present with increased macrophage infiltration, especially around renal tubules [29]. Macrophage accumulation closely associates renal injury and appears to worsen inflammation, a link that may be critical in the progression of age-related kidney diseases [30]. Consistent with results from renal tubular epithelial cells, we observed that Lck modulation also regulates leptin-induced inflammation in macrophages (Fig. 4B, C). Therefore, aging-induced hyperleptinemia may cause further inflammation via Lck activation in macrophages that infiltrate in the kidney.

Cumulative evidence highlights leptin’s involvement in the pathogenesis of age-related renal inflammation. Specifically, Lck inhibition lowers leptin-induced inflammation in renal tubular epithelial cells and macrophages via STAT3 and NF-κB signaling pathways (Fig. 4D).

In conclusion, we demonstrated a novel function for Lck in leptin-induced inflammatory signaling among renal tubular epithelial cells and macrophages. This study is also the first to provide evidence for the role of LepR short isoform in initiating Lck-STAT3/NF-κB pathway signaling transduction. Furthermore, we showed that increased Lck expression and hyperleptinemia interact to induce inflammation and accelerate renal aging. Importantly, our findings support the implementation of a therapeutic anti-aging strategy focused on blocking hyperleptinemia-induced Lck activity in peripheral and immune cells.
